# Controlled Magnesium Release and Nutritional Effect of a Novel Metal–Organic Framework on Plants

**DOI:** 10.1021/acs.cgd.5c00080

**Published:** 2025-06-23

**Authors:** Samuel Morales-Cámara, Lourdes Cardona-Carrascosa, Pablo Salcedo-Abraira, Antonio Rodríguez-Diéguez, Sara Rojas

**Affiliations:** 16741 University of Granada, Av/Fuente nueva s/n, 18071 Granada, Spain

## Abstract

Fertilizer application is a necessary action in modern agriculture to meet the global food demand. Modern agriculture must enhance crop production while minimizing the amounts of used fertilizers to ensure environmental safety without compromising their effectiveness. One promising approach to achieve this ambitious goal is using controlled-release fertilizers, which provide an effective amount of active ingredients prolonged over time while reducing the losses caused by leaching into the soil. In this work, a novel metal–organic framework (MOF) [Mg_2_(C_3_H_7_O_5_P)_2_(H_2_O)_4_]·H_2_O, named GR-MOF-27, based on the antibacterial agent fosfomycin (FMC) and magnesium is reported. First, its crystal structure was determined by single-crystal X-ray diffraction, and then its stability in aqueous media was studied. The results showed a prolonged two-step release of Mg^2+^ ions (release rates of 26% in 4 h and 63% in 7 d), which fits to pseudo-first-order kinetic (first 4 h) and pseudo-second-order (from 4 h to 7 d) release models. Since the metal precursor (magnesium sulfate) is normally used as a fertilizer, the nutritional effects of GR-MOF-27 and MgSO_4_ were evaluated and compared, obtaining the result that GR-MOF-27 improves plant growth by increasing shoot, root, and dried weight by 10.5, 11.0, and 13.1%, respectively. Additionally, plants treated with GR-MOF-27 showed an increase in the uptake of important macronutrients, such as 64.9% of Mg and 57.4% of P, demonstrating the benefits of using MOFs as slow-release fertilizers.

## Graphical abstract

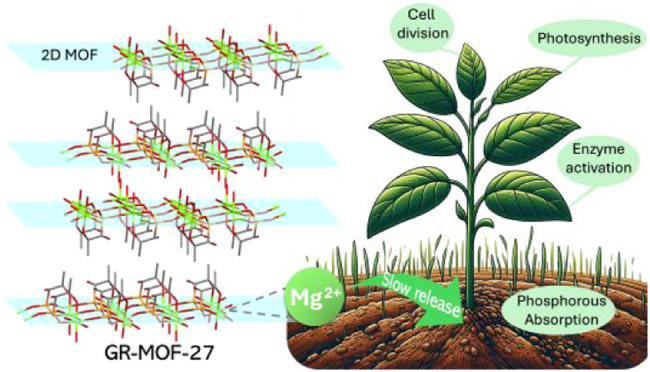

## Introduction

The growing population and the intensive use of lands make fertilizers essential to produce high-yield crops to meet global food requirements. Among the currently used fertilizers, inorganic fertilizers provide essential nutrients such as nitrogen (N), phosphorus (P), potassium (K), calcium (Ca), and magnesium (Mg). These elements are classified as macronutrients because plants require them in substantial quantities for proper growth and essential physiological processes.^1^ Particularly, Mg is a central core of chlorophyll, a molecule that captures the light energy to transform CO_2_ into carbohydrates during photosynthesis. It is also a cofactor for many enzymes involved in carbohydrates’ metabolism and synthesis of nucleic acids and proteins. Besides, Mg regulates the uptake of other essential elements for plant growth, like N, P, and Ca. Therefore, Mg is critical to maintain cellular functions and support the healthy development of plants.^2^ Mg deficiency (MGD) produces an early visible symptom in plants called interveinal chlorosis (leaves turn yellow due to the lack of chlorophyll degradation), growth retardation, reduction of biomass formation, and higher susceptibility to environmental stresses.^3,4^

Mg is considered an abundant element in the earth’s crust and can be found in minerals and rocks (i.e., dolomites or CaMg­(CO_3_)_2_). However, intensive Mg removal by high-yielding fertilizer-responsive cultivars and accelerated Mg leaching along with soil acidification continuously deplete indigenous exchangeable Mg reserves.^5^ Incidence of MGD or approaching deficient levels is expanding in most production systems.^6^ In agriculture soils, MGD is corrected using magnesium-based fertilizers, such as magnesium sulfate (MgSO_4_), by directly applying them in the solid from where they are quickly dissolved, releasing Mg^2+^ ions immediately available for plant uptake.^5^ However, the rapid release of Mg^2+^ can also be an inconvenience due to the easy leaching out during irrigation and rainfalls, reducing its availability to plants and polluting groundwater.

Slow-release fertilizers can prevent nutrient leaching, providing a sustained nutrient input prolonged in time. This approach enhances fertilizer efficiency, while reducing both the cost and environmental impact.^7^ Among all of the proposed materials, metal–organic frameworks (MOFs) have emerged as promising for slow-release agrochemical delivery.^8^ MOFs are an outstanding class of crystalline materials based on organic linkers coordinated to metallic centers, resulting in potentially porous structures.^9^ They have emerged as attractive materials for agrochemical applications due to their interesting properties such as large specific surface areas and pore volumes (which contribute to their exceptional sorption capacities), easily functionalizable cavities where specific host–guest interaction may occur, and the possibility of incorporating healthy or even active constituents, some of which are commercially available. Particularly, MOFs have been studied as agrochemical release agents through two different approaches, by their encapsulation as guest molecules because of their porosity, or by the release of their constituents.^8,10^ The first MOF described as a slow-release fertilizer was OPA-MOF (OPA: oxalate-phosphate-amine), which is a microbially induced slow-release N and P fertilizer.^11^ After a decade, only 23 studies (according to the Web of Science November 2024, “metal-organic framework”, “fertilizer”) have reported the use of MOFs as fertilizers and none of them as MGD agents.

Thus, we wish to take an additional step toward the application of MOFs in agriculture, and hence, the synthesis and full characterization of a novel two-dimensional MOF (namely, GR-MOF-27) based on Mg^2+^ and the antibacterial agent fosfomycin (FMC) is reported. This study investigates GR-MOF-27 as a Mg^2+^ release agent, specifically assessing its impact on plant growth. By achieving an Mg-controlled release, MOFs can contribute to both sustainable crop management and enhanced nutrient uptake efficiency.

## Results and Discussion

### Synthesis, Scale-up, and Characterization

A Mg-based two-dimensional-MOF (2D-MOF) was prepared by reacting FMC and MgSO_4_ in a 1:2 molar ratio in an aqueous solution at 95 °C (see Supporting Information-SI, Section S1). After 24 h, high-purity crystals of GR-MOF-27 were obtained. Scanning electron microscopy (SEM) images showed well-faceted flat hexagonal microcrystals with the size of ca. 30–50 μm (SI, Figure S1), suitable for single-crystal X-ray diffraction (SCXRD) (SI, Section S1&S2). GR-MOF-27 crystallizes in the monoclinic *C*2/*c* space group with the formula [Mg_2_(C_3_H_7_O_5_P)_2_(H_2_O)_4_]·H_2_O. Surprisingly, the epoxide ring of FMC is hydrolyzed by an in situ reaction to obtain 1,2-dihydroxypropyl phosphonic acid (Figure 1), yielding 75% of the *R*,*R’* isomer and 25% of the *S*,*S’* one, coordinating both the Mg atoms in a disordered way (SI, Figure S2).

**Figure 1:**
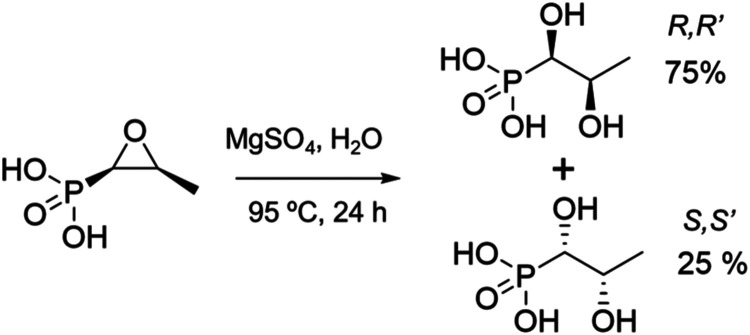
In situ FMC ring opening to yield 1,2-dihydroxypropyl phosphonic acid.

The secondary building unit (SBU) can be described as an MgO_6_ octahedra. Each Mg is coordinated to two water molecules and three linker molecules (SI, Figure S3). One ligand is coordinated through a monodentate phosphonate group and one hydroxyl group and the other two by monodentate phosphonate groups, all of them having d­(Mg···O) values between 1.95(4) and 2.183(9) Å (SI, Table S2).

On the other hand, each ligand coordinates 3 different Mg atoms: one chelated by the hydroxyl and monodentate phosphonate groups and the other two by just the other two oxygen atoms of the phosphonate group. Note here that in the case of the *R,R’* ligand the coordination of the hydroxyl is through the O5, while for the *S,S’* isomer, it is by the O4 (SI, Figure S2). The coordination between the Mg and the ligand creates a 2D layer along the (100) plane with all of the aliphatic chains on one side and all of the water molecules (both coordinated and free) on the other side (Figure 2). These layers are stacked in opposite ways, establishing both van der Waals interactions (through the aliphatic chains) and hydrogen bonds (between the coordinated and free water molecules) (SI, Figure S4). The unit cell contains 4 of these layers along the *b* and *c* axes.

**Figure 2:**
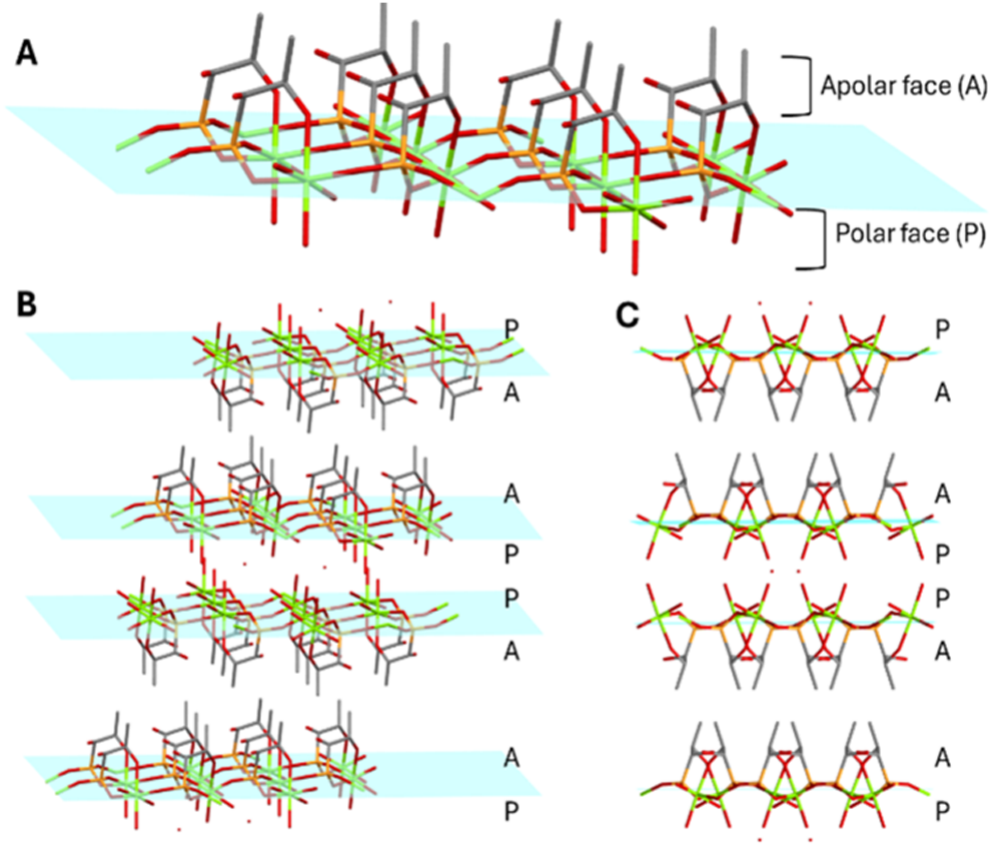
GR-MOF-27 2D plane exhibiting two distinct faces, polar and apolar (A). In the crystal structure, each plane is stacked with polar faces aligning with polar faces and apolar faces aligning with apolar faces along the *b* (B) and *c* axes (C). Carbon: gray; oxygen: red; magnesium: green; phosphorus: orange. Hydrogen atoms and disordered ligands are not shown here for simplicity.

Remarkably, the syntheses of GR-MOF-27 were successfully scaled up 20 times (from 0.071 to 1.42 mmol of FMC) from solvothermal conditions in closed vials (reaction volume 5.2 mL) to a round-bottom flask (104 mL), obtaining up to ∼0.25 g of solid (36% yield) in a single reaction. The characteristic crystalline phase of all compounds was identified in the scaled-up bulk samples by comparing the location and intensity of the main Bragg reflections with those of the crystalline structure resolved by SCXRD (Figure 3). To check the phase purity, Le Bail fitting was carried out using the unit cell parameters of GR-MOF-27 (SI, Figure S5). Fourier transformed infrared (FT-IR) spectra confirm the presence of coordinated FMC as there is an important shift of the phosphonate bands (P=O, st and P–O, st) from 1096 and 1122 cm^–1^ in FMC^12^ to 1074 and 1099 cm^–1^ for GR-MOF-27 (SI, Figure S6). The thermogravimetric analysis (TGA) curve shows an initial weight loss (from RT to 270 °C) attributed to the departure of five water molecules (20.72%) and a second one (at 310 °C) corresponding to the decomposition of the compounds by the oxidation and departure of the linker (SI, Figure S7).

**Figure 3:**
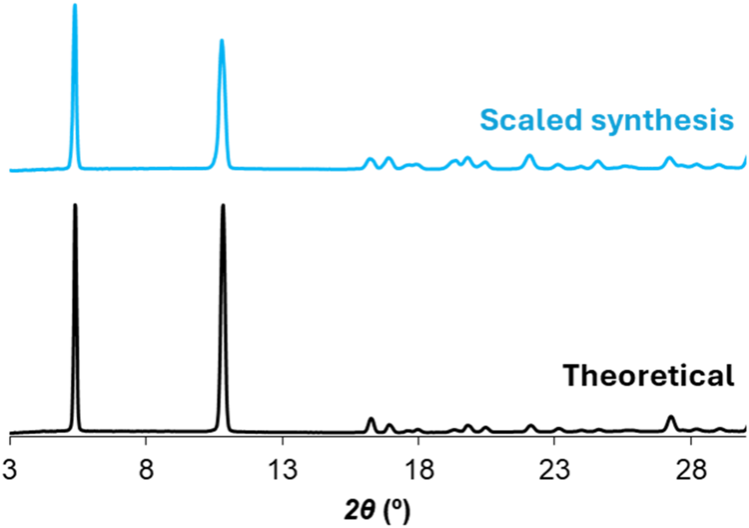
Powder X-ray diffraction (PXRD) patterns of scaled synthesis (blue) compared with the theoretical diffractogram obtained from the SCXRD data (black) of GR-MOF-27.

### Stability Studies

As previously mentioned in the introduction, Mg-based agrochemicals are typically applied as solid formulations in agricultural fields, where the release of Mg is primarily driven by its solubilization in water from soil humidity, irrigation, or rainfall.^13^ In this regard, the potential of GR-MOF-27 as an agrochemical was assessed by evaluating its chemical and structural stability in water.

The chemical robustness in solution was investigated by inductively coupled plasma atomic emission spectroscopy (ICP-OES) by means of the release of active Mg^2+^ cations for 21 d. Note here that, according to the Pourbaix diagram of Mg, at the studied pH (6.5) the potentially released metal exists in solution as an Mg^2+^ cation.^14^ Further, it should be noted that the Mg^2+^ release is here performed under simulated realistic conditions since the used pH value is found in the majority of soils (5.5–7.5).^15^ The results showed a two-step release of Mg^2+^, with an initial fast step (26% in 4 h) followed by a continuous release of the metal with time (up to 63% in 7 d) when GR-MOF-27 is suspended in water (Figure 4A). Regarding structural stability, the crystal structure of GR-MOF-27 is maintained after its suspension in water for 7 days, as demonstrated by the PXRD patterns (Figure 4B). After 21 days no remaining solid was recovered. Considering all of the data, we can suggest that the release of Mg^2+^ is driven by the degradation of the framework.

**Figure 4:**
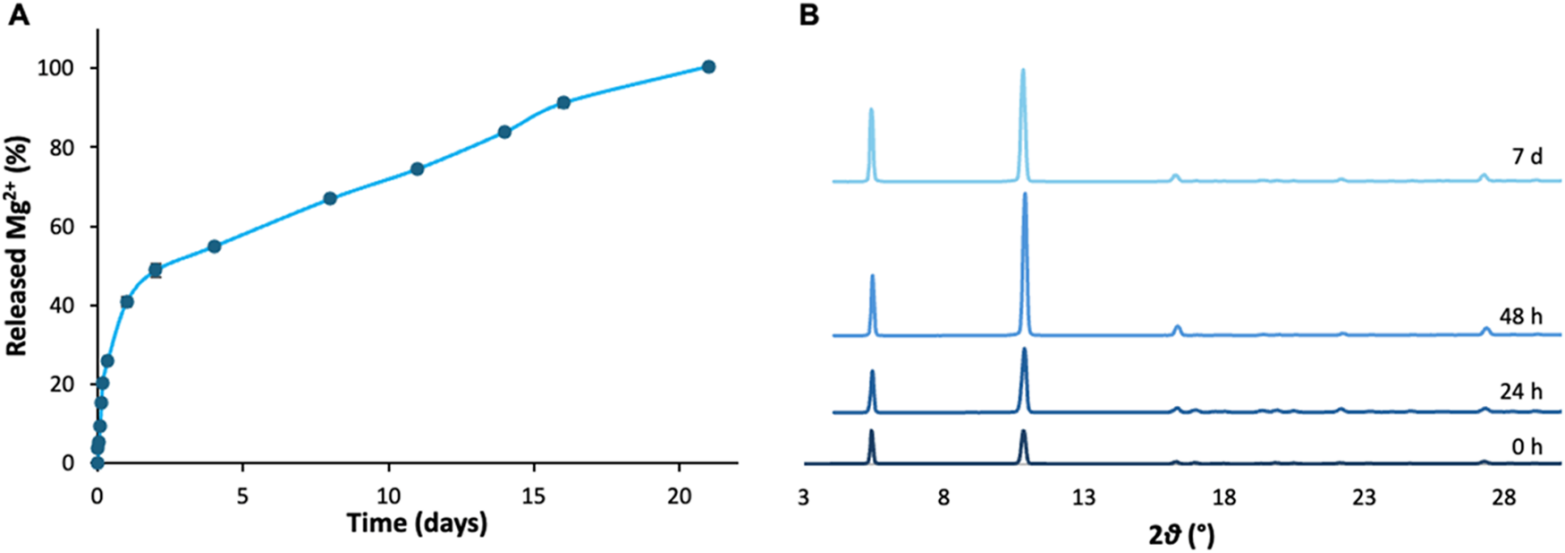
Mg^2+^ leaching from GR-MOF-27 (A) and PXRD patterns over time when suspended in water at room temperature (B).

At this point, in order to gain an understanding of and make MOFs more efficient for practical uses, the first and second steps of the Mg^2+^ release were satisfactorily fitted to a pseudo-first-order (PFO) kinetic (first step, 4 h) and pseudo-second-order (PSO) models (from 4 h to 7 d), respectively. Note that the kinetic study was performed under continuous stirring to exclude the external diffusion process around the particles. The initial release was successfully fitted to a PFO model, which is applied to describe the release of water-soluble compounds from porous matrices.^16^ The Mg^2+^ release fits to this model with a regression factor (*R* ^2^) > 0.998 (SI, Figure S8). Thus, the Mg^2+^ release could be explained by the equation $\ln\left(q_{e}-q_{t}\right)=\ln\left(q_{e}\right)-K_{1}\cdot t$ , where *q*_e_ is the maximum release capacity (mg·g^–1^), *q*_*t*_ is the release capacity (mg·g^–1^) in that time (*t*), and *K* is the release constant for PFO (h^–1^). The second step fitting to a PSO model and an *R* ^2^ > 0.996 (SI, Figure S9) is related to the rate of the ion exchanged on the surface of the material.^17,18^ Here, the desorption data can be explained by $\frac{t}{q_{t}}=\frac{1}{K_{2}\cdot q_{e}^{2}}+\frac{t}{q_{e}}$ , where *K*_2_ is the release constant for PSO (g·mg^–1^·h^–1^).

It is important to notice that a gradual release of the active ingredients is essential for the final application, which involves the controlled release of agrochemicals in fields. Although it is not an easy task, the findings of this work were contextualized within the field of Mg-based agrochemicals, and a comparison was made using the kinetic data obtained and previous reports using similar experimental conditions (25 °C; pH = 6.5; stirring) (SI, Table S3). For instance, the nanofertilizer nDPF2 exhibited a 12.31% Mg release over 8 d with *K* = 2.764 h^–1^,^19^ while GR-MOF-27 showed 26% in 4 h with *K* = 0.122 h^–1^, and 63 and 100% in 7 and 21 d with *K*_2_ = 0.005 g·mg^–1^·h^–1^. These results denote that while GR-MOF-27 releases a higher total amount of Mg, the release rate is slower compared to that of nDPF2.

Although this novel material is still far from its application in the field, our work presents an Mg-release agent with a lower calculated kinetic constant and a release percentage in the range of other previously reported materials, demonstrating the potential of MOFs as Mg fertilizers.

### Evaluation of the Nutritional Effect of GR-MOF-27

Italian raygrass () was used to evaluate the effectiveness of GR-MOF-27 as a nutrient. First, the active GR-MOF-27 concentration was determined by submerging previously germinated seeds (3 d) with various GR-MOF-27 concentrations and comparing with a control group (water). The tested GR-MOF-27 concentrations (91, 910, and 1820 ppm) were selected based on the Mg content in the MOF (10, 100, and 200 ppm of Mg). After growing the plants for 7 d, 91 ppm of GR-MOF-27 (10 ppm of Mg) was considered as the optimal concentration for growth (SI, Section S4 for further details). Importantly, this concentration is on the range of the recommended Mg rate in our fields (23.2 kg·hm^2^ or 2.9 mg per beaker) under severe soil Mg deficiency conditions.^20^ Note that when higher concentrations of GR-MOF-27 were used (1820, 200 ppm of Mg content), the inhibition of plant growth was observed, which is probably related to the higher Mg^2+^ concentrations, as previously reported.^21^

Once the optimal GR-MOF-27 concentration was determined and in order to assess the importance of these results, the activity of GR-MOF-27 was compared with MgSO_4_, which is normally used as the fertilizer to correct Mg deficiency and also is the metal precursor for GR-MOF-27. During this study, an equivalent content of Mg (10 ppm) was used for both compounds, GR-MOF-27 (91 ppm) and MgSO_4_ (41 ppm). When GR-MOF-27 was used, all of the measured parameters (shoot and root lenght, and dried weight) were significantly better than when using MgSO_4_ (positive control) or water (negative control) (Figure 5). GR-MOF-27 (91 ppm) was able to significantly (*p*-value <0.05) increase the shoot and root length and dried weight (10.95 ± 2.2, 8.76 ± 2.45 cm, 2.75 ± 0.18 mg) when compared with MgSO_4_ (9.80 ± 1.98, 7.80 ± 1.91, 2.39 ± 0.09) and water (9.16 ± 1.78, 7.50 ± 2.12 cm, 2.34 ± 0.15 mg). The increment observed for shoot and root length, and dried weight is ca. 10.5, 11.0, and 13.1%, respectively, when compared with MgSO_4_, and *ca*. 16.4, 14.4, and 15.0%, respectively, when compared with water. Therefore, we can argue that GR-MOF-27 with a slow release of their components produces an improved fertilizer effect in the than the commonly used MgSO_4_ at equivalent magnesium content.

**Figure 5:**
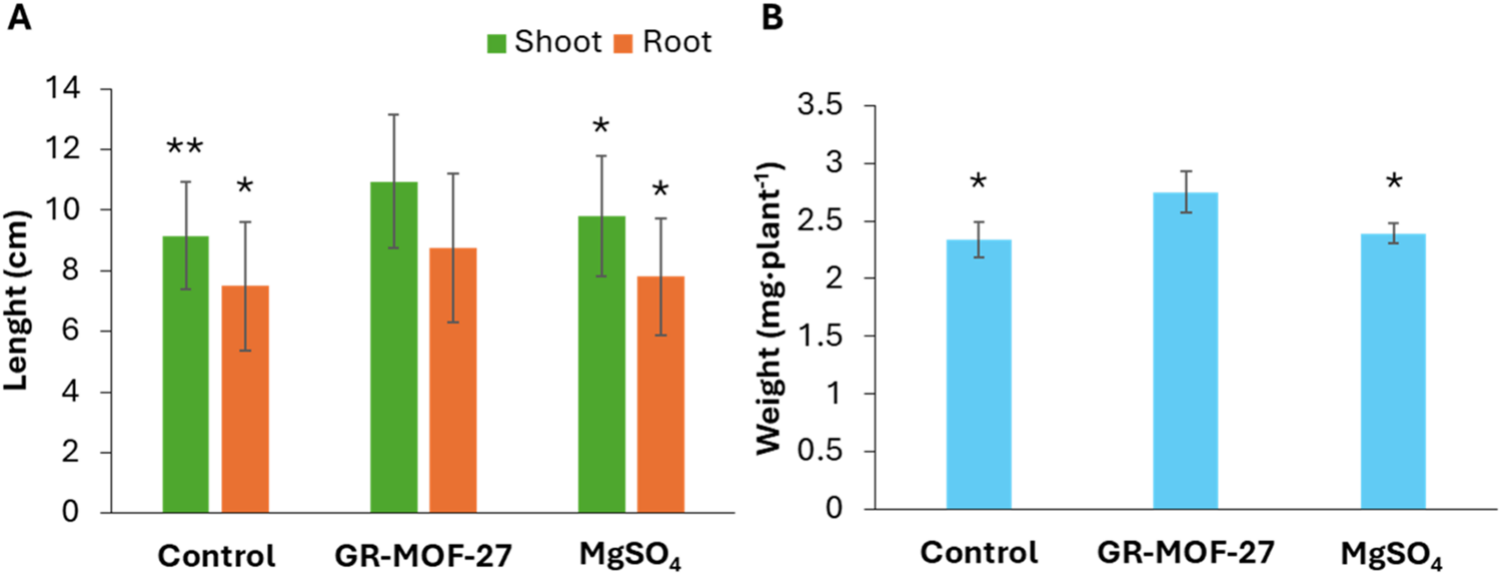
Effect of GR-MOF-27, MgSO_4_ (commercially used fertilizer), and water (control) on shoot and root length (cm) (A) and on the dried weight per plant (mg) (B). Values of GR-MOF-27 were compared with control (water) and MgSO_4_ to identify significant differences; ANOVA test was performed where *p*-value *<0.05 and **<0.001.

Finally, the comparison of the elemental compositions of the plants treated with GR-MOF-27 at the effective concentration and water (control) supported these results, showing that there is an increment of 64.9 and 57.4% in the uptake of Mg and P, respectively (SI, Figure S11). Thus, the efficacy of GR-MOF-27 as a fertilizer might be related to both the slow release of their components, preserving the environment, and also the simultaneous adsorption of Mg and P, as Mg regulated the uptake of other essential elements by plants.

## Conclusions

A novel 2D-MOF based on magnesium and fosfomycin has been synthesized and fully characterized. GR-MOF-27 exhibited high stability on water medium for up to 21 days, releasing Mg^2+^ at a slow rate following a two-step release process (26, 63, and 100% in 4 h, 7, and 10 days, respectively). Mathematical models indicated that the first and second steps of the Mg^2+^ release fit the PFO and PSO model, respectively. Regarding the bioactivity, GR-MOF-27 enhanced the overall length and biomass of compared to the traditional fertilizer MgSO_4_, with the shoot and root length, and dried weight increased by 10.5, 11.0, and 13.1%, respectively. The compositional analysis of plants showed an increment of nutrient uptake (64.9% of Mg and 57.4% of P). Therefore, GR-MOF-27 produces a better nutritional effect than traditionally used fertilizers. This work provides an original approach to employ MOFs as Mg slow-release systems, improving the activity of traditional Mg-based fertilizers and making agriculture practices more efficient and sustainable.

## Supplementary Material



## Notes

Materials and methods, additional crystallographic data, GR-MOF-27 SEM, Le Bails fitting, FT-IR, TGA, additional information about the kinetic release model fitting, and additional nutritional plant effect information and comparison with other fertilizers (PDF)
