# Supplementary material for: Controlled Magnesium Release and Nutritional Effect of a Novel Metal–Organic Framework on Plants
Source: Cryst Growth Des. 2025 Jun 23;25(13):4685–9. doi: 10.1021/acs.cgd.5c00080 (PMC12232427; doi:10.1021/acs.cgd.5c00080)
Supplement: Supplementary file 1 [file cg5c00080_si_001.pdf]

Controlled Magnesium Release and Nutritional Effect on Plants of a Novel Metal-Organic Framework

Samuel Morales-Cámara, Lourdes Cardona-Carrascosa, Pablo Salcedo-Abraira, Antonio Rodríguez-Diéguez,* and Sara Rojas*

Department of Inorganic Chemistry, Faculty of Science, University of Granada. Av. Fuente nueva s/n, 18071 Granada, Spain. E-mail: srojas@ugr.es, antonio5@ugr.es

Supporting information

Table of contents

S1. Materials and methods……………………………………………………S2

S2. Crystallographic studies and material characterization………………...S4

S3. Aqueous stability and release kinetic studies……………………………S7

S4. Plant nutritional effect……………………………………………………S10

S5. References…………………………………………………………………S13

S1. Materials and methods

All chemicals were commercially obtained and used without further purification. Fosfomycin disodium salt (FMC, TCI, 98%), magnesium sulphate anhydro ($MgSO_4$, Labkem, 95%), deionized water (pH = 6.4) as reaction solvent, and ethanol (96%, VWR Chemical) for washing.

Synthesis of GR-MOF-27. 0.071 mmol (13 mg) of FMC were dissolved in 2.6 mL of deionized water. In a separate vial, 0.143 mmol (17.2 mg) of $MgSO_4$ were dissolved in 2.6 mL of deionized water and dropped to the FMC solution. The resulting solution was kept under hydrothermal conditions in a closed glass vial (volume 26 mL) and heated at 95 °C for 24 h. White X-ray quality single crystals were obtained, filtered off and washed with 5 mL of ethanol 3 times. Yield (based on metal): 36%. Anal. Calcd for $[Mg_2(C_3H_7O_5P)_2(H_2O)_4]\cdot H_2O$ (%): C (16.13), H (5.41); found: C (15.85), H (7.25); MW = 446.8 g·mol^{-1}. Residue after thermal treatment: 52.06%. It was not possible to identify the nature of the final residue using PXRD due to its amorphous state.

Scale-up synthesis. The synthesis was scaled 20 times. 1.42 mmol (0.26 g) of FMC were dissolved in 52 mL of deionized water. In a separate vial, 2.86 mmol (0.34 g) of $MgSO_4$ were dissolved in 52 mL of deionized water and dropped to the fosfomycin solution. The resulting solution was heated at 100 °C under reflux and stirring (800 rpm) overnight. White powder was obtained, filtered off and washed with 5 mL of ethanol 3 times. Yield (based on metal): 36%.

Physicochemical characterization. Elemental analyses (EA) were carried out on a Thermo Scientific analyzer model Fash 2000. The Fourier transform infrared (FT-IR) spectra, measured on powdered samples in an attenuated total reflectance (ATR) mode, were recorded on a Bruker Tensor 27 FT-IR and Opus data collection program. Powder X-ray diffraction (PXRD) patterns of all samples were collected in a BRUKER D8 ADVANCE equipment, where the routine conditions were from 3 to 30º (2θ) using a step size of 0.02° and scan rate of 30 s *per* step. Thermogravimetric analyses (TGA) were performed using a Mettler Toledo TGA/DSC STAR system under oxygen flow (20 mL min^{-1}) running from room temperature (RT) to 600 °C with a heating rate of 5 °C min^{-1}. Scanning electron microscopy (SEM) was carried out using a Hitachi S510 microscopy at 25 kV coupled with a SE detector of 7 nm at 25 kV at Centro de Instrumentación Científica, University of Granada. ICP-OES was done in a spectrometer Perkin Optima 7300DV and ICP-MS in a Perkin SCIEX NexION 300D, both performed at Servicios Centrales de Apoyo a la Investigación (SCAI), University of Málaga.

Single crystal structure determination. Large single-crystals were obtained for their structure resolution by single crystal X-Ray Diffraction. X-ray data collection was done at 296.15 K on a Bruker D8 Venture diffractometer using a photon detector equipped with graphite-monochromated MoKα radiation (λ = 0.71073 Å). The data reduction was performed with the APEX3 software[1] and corrected for absorption using SADABS.[2] Crystal structure was solved by direct methods using the SHELXT program[3] and refined by full-matrix least-squares on F2 including all reflections, using anisotropic displacement parameters. The occupation disorder in the alkyl chains was first refined free and then fixed to the found values All hydrogen atoms were located in idealized geometries, and included, as fixed contributions riding on attached atoms with isotropic thermal displacement parameter 1.2 times those of their parent atoms. The OLEX2 software[4] was used as a graphical interface. Crystallographic data for the reported structure have been deposited with the Cambridge Crystallographic Data Center CCDC under the number 2395438. Copies of the data can be obtained free of charge at http://www.ccdc.cam.ac.uk/products/csd/request.

Table S1. Crystallographic data and structure refinement details of GR-MOF-27.

**CCDC number**	2395438
**Empirical formula**	$C_6H_{24}Mg_2O_{15}P_2$
**Formula weight**	446.81
**Temperature/K**	296.15
**Crystal system**	monoclinic
**Space group**	*C*2/*c*
**a/Å**	33.9395(16)
**b/Å**	5.0776(2)
**c/Å**	10.4411(4)
**α/°**	90
**β/°**	105.871(2)
**γ/°**	90
**Volume/Å^3**	1730.74(13)
**Z**	4
**ρ_{calc}g/cm^3**	1.715
**μ/mm^{-1}**	0.398
**F(000)**	936.0
**Crystal size/mm^3**	0.08 × 0.04 × 0.001
**Radiation**	MoKα (λ = 0.71073)
**2Θ range for data collection/°**	4.992 to 57.362
**Index ranges**	$-43 \leq h \leq 45$,<br>$-6 \leq k \leq 6$,<br>$-14 \leq l \leq 11$
**Reflections collected**	5485
**Independent reflections**	2171 [R_{int} = 0.0485, R_{sigma} = 0.0414]
**Data/restraints/parameters**	2171/0/159
**Goodness-of-fit on F^2**	1.309
**Final R indexes [I>=2σ (I)]**	R_1 = 0.0859, wR_2 = 0.1760
**Final R indexes [all data]**	R_1 = 0.1101, wR_2 = 0.2251
**Largest diff. peak/hole / e Å^{-3}**	1.62/-0.69

S2. Crystallographic studies and material characterization

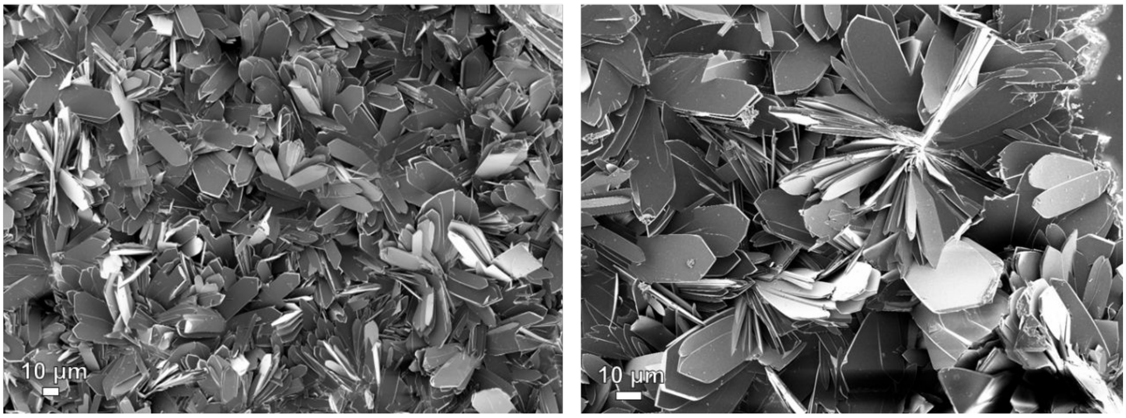

Figure S1. Scanning electron microscopy (SEM) images of the GR-MOF-27 crystals.

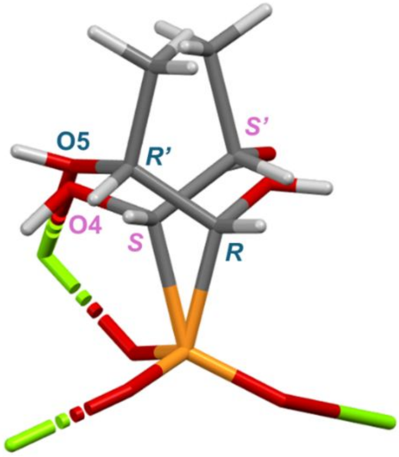

Figure S2. Coordination of the disordered ligands to the Mg atoms. Labels of the *R,R'* and *S,S'* isomers are in dark blue and pink, respectively. Carbon: grey, Oxygen: red, Hydrogen: white, Magnesium: green, Phosphorus: orange.

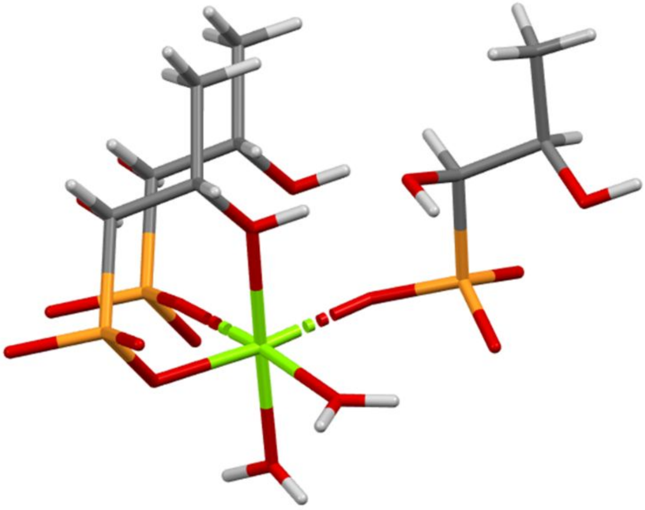

Figure S3. MgO_6 octahedra coordination. Only the *R,R'* isomer was represented in terms of clarity. Carbon: grey, Oxygen: red, Hydrogen: white, Magnesium: green, Phosphorus: orange

Table S2. Selected bond lengths of GR-MOF-27.

Atom	Atom	Length/Å	Atom	Atom	Length/Å
$Mg^{(1)}$	$O^{(1)}$	2.046(4)	$Mg^{(1)}$	$O^{(5A)2}$	2.183(9)
$Mg^{(1)}$	$O^{(1W)}$	2.072(4)	$Mg^{(1)}$	$O^{(4B)2}$	1.95(4)
$Mg^{(1)}$	$O^{(2)1}$	2.028(4)	$Mg^{(1)}$	$O^{(2W)}$	2.121(4)
$Mg^{(1)}$	$O^{(3)2}$	2.061(3)			

[1]+X,-Y,1/2+Z; [2]+X,-1-Y,1/2+Z

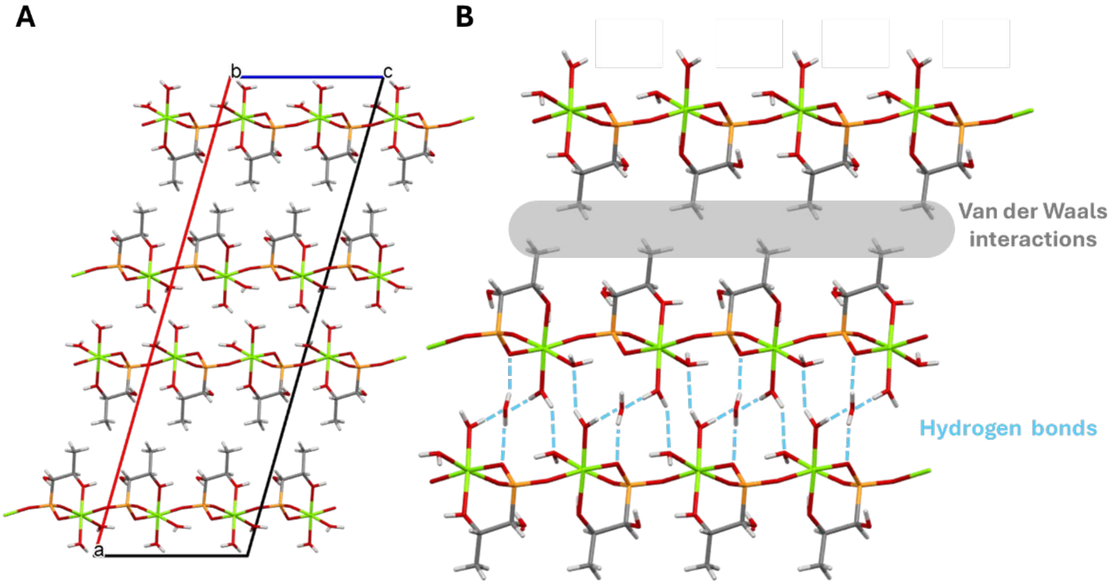

Figure S4. GR-MOF-27-unit cell is formed by four chains stacking on each other (A). One chain is stacked to another trough the methyl groups via van der Waals interactions, while it interacts with another chain trough hydrogen bonds (distance *ca.* 1.9 Å) established between coordinated water molecules and crystallization water(B). Carbon: grey, Oxygen: red, Hydrogen: white, Magnesium: green, Phosphorus: orange

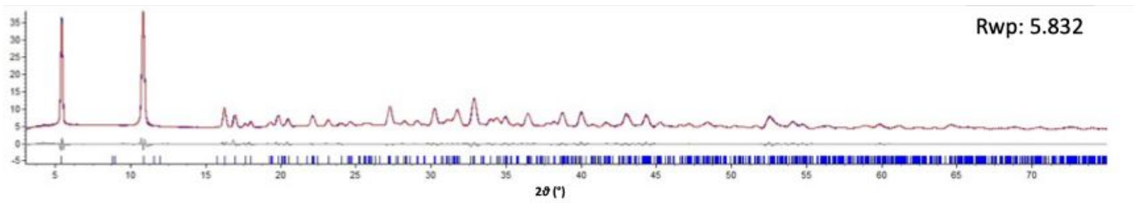

Figure S5. Le Bail fitting of GR-MOF-27.

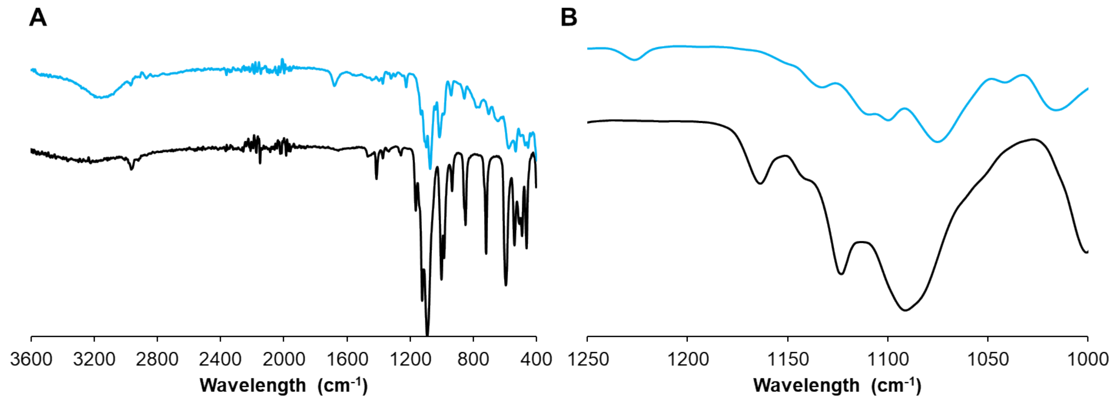

Figure S6. A) Fourier Transform Infrared (FT-IR) spectra of GR-MOF-27 (blue) compared with ligand (black). B) Displacement of the phosphate bands (P=O, st) in the MOF in comparison with the free linker.

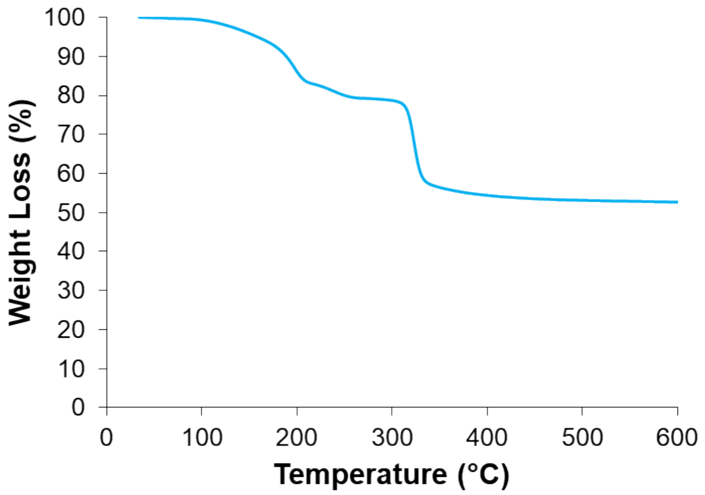

Figure S7. TGA of GR-MOF-27.

S3. Aqueous stability and release kinetic studies

The chemical stability of GR-MOF-27 in MilliQ water (pH = 6.5) at RT was checked by measuring the release of Mg^{2+} by ICP-OES. 20 mg of GR-MOF-27 was suspended in 20 mL of MilliQ water under stirring for 21 days, at different intervals of time (15, 30 min, 1, 2, 3, 4, 8 hours, 1, 2, 4, 8, 11, 14, 16 and 21 days) the suspensions were centrifuged (14000 rpm, 1 min), and half of the volume (mL) was replaced by fresh MilliQ water (always working under sink conditions). The removed liquid phase was analyzed by IPC-OES. The experiment was carried out in triplicate (n = 3), and the data obtained was analyses as the mean and standard deviation. These data were used to determine the release rate of Mg and, plotting Mg^{2+} release percentage versus time.

Additionally, the Mg^{2+} release data during the first 4 h was analyzed to fit four mathematical models of release kinetics: pseudo-first-order (PFO) and pseudo-second-order (PSO), zero order and Higuchi model, all of them amply used in adsorption but also in release kinetics. The PFO model is applied to describe the release of water-soluble compounds from porous matrices.[5] This model is widely used for large adsorption times when the system is close to equilibrium. The PSO is usually associated with processes where the rate of direct adsorption/desorption process (seen as a chemical reaction) controls the overall sorption kinetics.[6] Thus, the rate of the ion exchanged on the surface is responsible for the release kinetics. Higuchi model defines the short time behavior of the release/adsorption of a disperse adsorbent from a homogeneous matrix.[7] Finally, the zero-order model is often used to describe the adsorbate dissolution of several types of modified release matrixes, as well as matrices with low soluble adsorbate.[8]

The equations describing these models are:

PFO

$$\frac{dq(t)}{dt} = K_1 \cdot (q_e - q_t) \qquad \text{Equation 1}$$

and its integrated and linearized form:

$$Ln(q_e - q_t) = Ln(q_e) - K_1 \cdot t \qquad \text{Equation 2}$$

where q_e is the maximum release capacity in the equilibrium ($mg \cdot g^{-1}$), q_t is the release capacity in that time ($mg \cdot g^{-1}$), and K_1 is the release constant for PFO (h^{-1}).

PSO

$$\frac{dq(t)}{dt} = K_2 \cdot (q_e - q_t)^2 \qquad \text{Equation 3}$$

and its integrated and linearized form:

$$\frac{t}{q_t} = \frac{1}{K_2 \cdot q_e^2} + \frac{t}{q_e} \qquad \text{Equation 4}$$

where q_e is the maximum release capacity in the equilibrium ($mg \cdot g^{-1}$), q_t is the release capacity in that time ($mg \cdot g^{-1}$), and K_2 is the release constant for PSO ($g \cdot mg^{-1} \cdot h^{-1}$).

Zero Order

$$q_t = K{\cdot}t \qquad \text{Equation 5}$$

where t is the time (h), q_t is the release capacity in that time (mg·g^{-1}), and K is the zero-order kinetic constant (mg·g^{-1}·h^{-1}).

Higuchi model

$$q_t = K{\cdot}\sqrt{t} \qquad \text{Equation 6}$$

where t is the time (h), q_t is the release capacity in that time (mg·g^{-1}). K is the release constant for Higuchi model (g·mg^{-1}·h$^{-1/2}$).

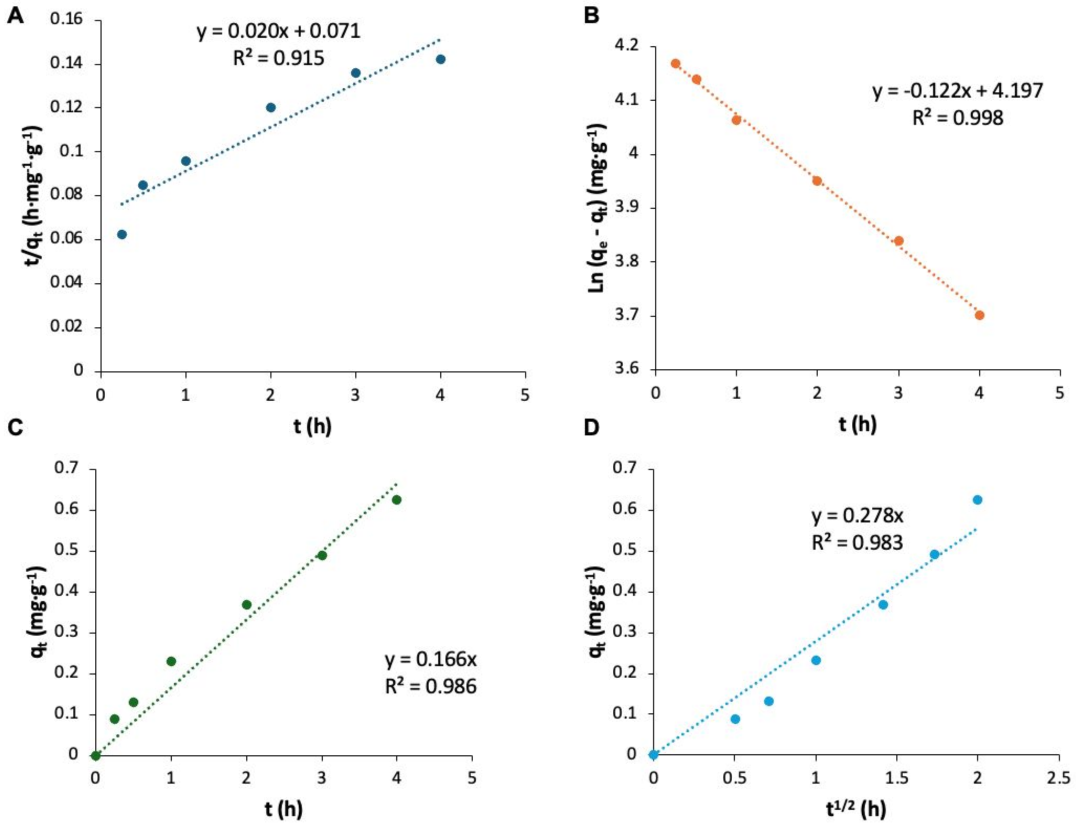

Figure S8. Fitting data for the release of Mg^{2+} from GR-MOF-27 during the first 4 hours using a A) PSO, B) PFO, C) Zero-order, and D) Higuchi model. The equation and the goodness of fit (R^2) of each model are indicated.

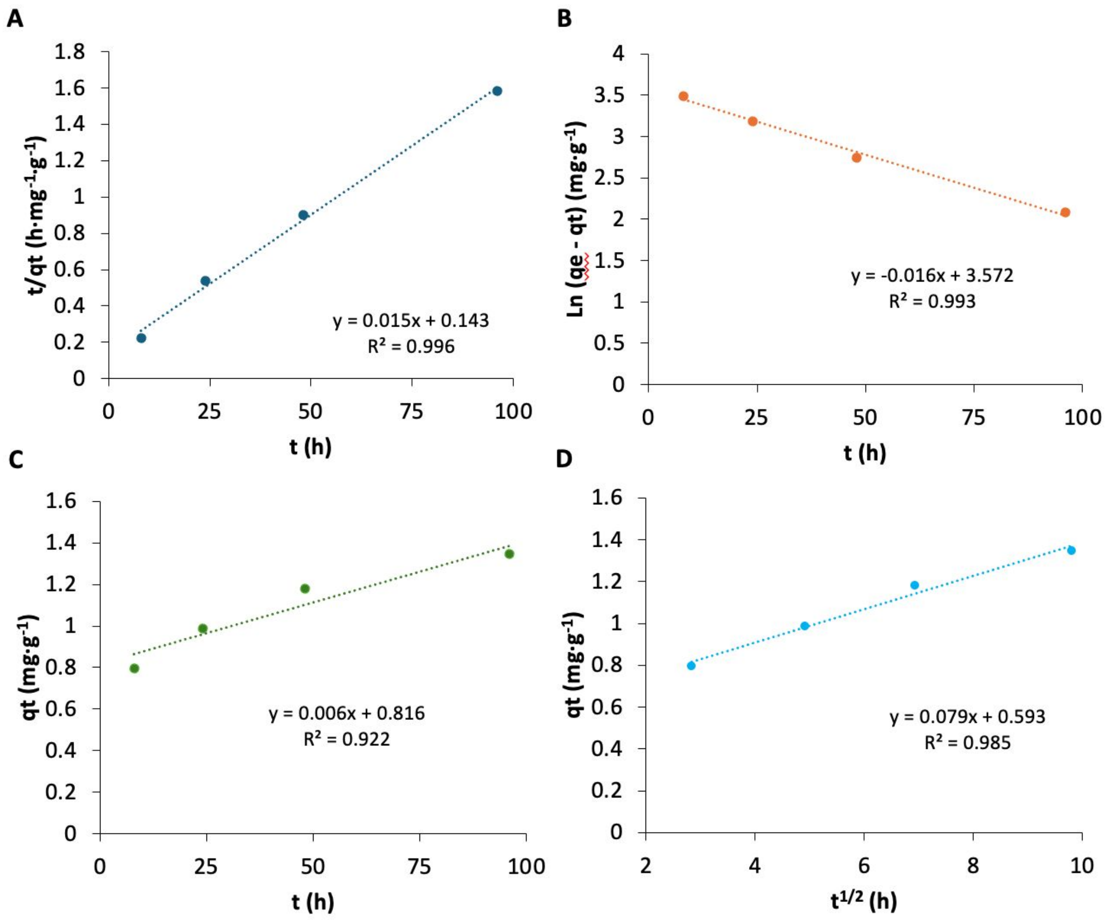

Figure S9. Fitting data for the release of Mg^{2+} from GR-MOF-27 from 4 h to 7 d using a A) PSO, B) PFO, C) Zero-order, and D) Higuchi model. The equation and the goodness of fit (R^2) of each model are indicated.

In parallel, the structural stability was determined by suspending 10 mg of compound in 5 mL of deionized water at RT under stirring for 21 days. At the different suspension times, the suspensions were centrifuged (1 min, 14000 rpm), the solids were recovered and analyzed by PXRD to check the crystallinity of the structure.

S4. Plant nutritional effect

The nutritional effect of GR-MOF-27 was studied based on its ability to release Mg over time, as previously reported using other Mg-based agrochemicals (Table S3).

Table S3. Comparison of Mg loading and release using already reported Mg-agrochemicals.

Agrochemical	Mg (wt%)	Release conditions	Released Mg (%), Time		Ref.
			**Released Mg (%), Time (h,d)**	**Kinetic model, *K***	
$Mg(SO_4)\cdot 7H_2O$	9.86	25°, water	*ca.* 70, 1 d		9
MgO	60.30	25°, water	2.3, 1 d		9
$Mg(OH)_2$	41.68	25°, water	2.1, 1 d		9
$MgCl_2\cdot 6H_2O$	11.96	25°, water	93, 10 d		9
$Mg(NO_3)_2\cdot 6H_2O$	9.48	25°, water	96, 10 d		9
nDPF2	5.24	water	12.3, 8 d	power function, 2.764 h^{-1}	10
nDPF1	4.51	water	17.17, 8 d	power function, 6.38 h^{-1}	10
GR-MOF-27	10.88	RT, water	26, 4 h<br>63, 7 d<br>100, 21 d	(4 h) PFO, 0.122 h^{-1}<br>(8 h – 7d) PSO, 0.005 $g\cdot mg^{-1}\cdot h^{-1}$	This work

The nutritional effect was studied in terms of seedling growth. *Lolium multiflorum* (Italian ray-grass), purchased from Semillas Batlle (Barcelona, Spain), was used as model plant in this study due to its rapid germination and growth rate, as well as its ability to grow in liquid media under hydroponic conditions.[12]

Seeds were kept in a dry place in the dark under room temperature before their use. Initially, seeds were germinated on wet paper under dark conditions at 25 °C for three days. After 3 days, seedlings of uniform size were used in the nutritional test. Nutritional tests consist of exposing plants to the different compounds suspended in tap water and allowing them to grow under ambient humidity and temperature conditions, with an average day/night photoperiod of 15-9 h, for 7 days. After the growth period, root and shoot lengths were measured by photographing plants and analyzing the images using ImageJ software for precise measurements. The shoot and root lengths are presented as the mean and standard deviation of all sample exposed to the same treatment (n = 45). The plant weight was also evaluated drying samples in an oven at 95 ºC for 2 hours. The dried weight values are reported as the mean and standard deviation of three replicates, with each replicate consisting of 15 plants that were weighted together. The statistical analysis was conducted using an ANOVA test, with significance levels set at p-values of 0.001, 0.01, and 0.05.

First, the optimal concentration of GR-MOF-27 in *L. multiflorum* growth was determined. Aqueous suspensions (10 mL) of GR-MOF-27 with different concentrations (91, 910 and 4550 ppm, corresponding to 10, 100 and 200 ppm of Mg, or 0.9, 9.1 and 18.2 mg per beaker) were tested. 50 mL beakers (12.56 cm^2 surface area) were used for each different concentration, with a total *ca.* 15 previously germinated seeds. Note here that our experiments are in the average of the recommended Mg rate is 23.2 $Kg\cdot ha^{-1}$ (or 2.9 mg per beaker) under severe soil Mg deficiency conditions.[11] In parallel, a negative control with only water was performed. Various parameters (shoot and root length, and dry weight) were measured to evaluate the effect in plants of the different concentrations of GR-MOF-27 comparing with control. GR-MOF-27 at 91 ppm significantly increases the shoot, root and weight in *ca.* 16.4, 14.1 and 15.0%. However, 910 ppm

does not increase the shoot length and weight, producing a significant decrease in roots *ca.* 19% (**Figure S10**). The highest concentration tested (182 ppm of GR-MOF-27; 200 ppm of Mg) inhibit drastically the plant grow, probably related with higher Mg^{2+} doses as previously reported.[12] Thus, 91 ppm was considered the optimal concentration of *L. multiflorum* development.

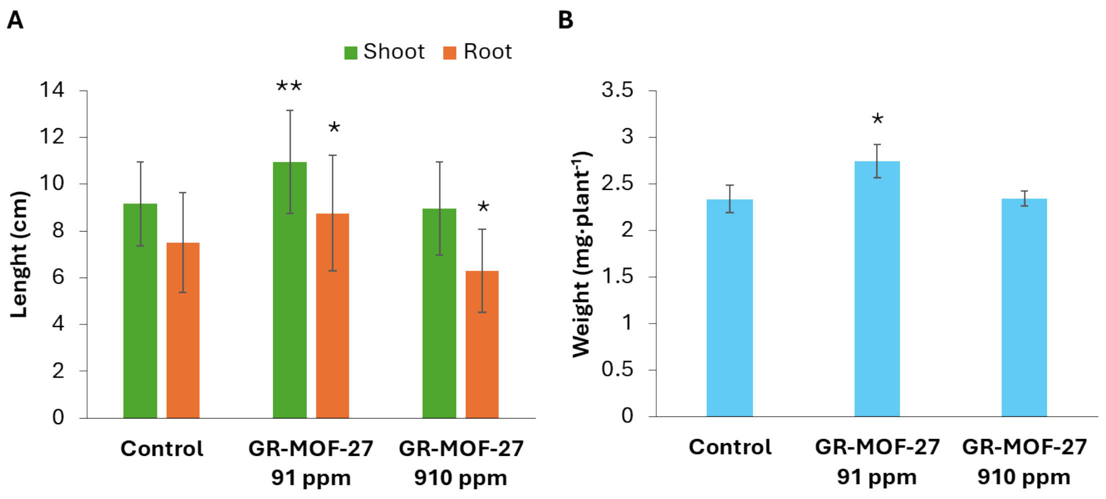

Figure S10. Effect of different concentration of GR-MOF-27 on (A) shoot and root length (cm), and (B) weight per seed (mg). Average and standard deviation is represented. Values of each treatment were compared to identify significant differences; ANOVA test was performed where *p*-value *< 0.05; **<0.001.

The elemental composition of plants after growing with the optimal concentration of GR-MOF-27 was analyzed by ICP-MS to determine the effect of GR-MOF-27 in the nutrient uptake. In particular, Mg, Ca and P were analyzed by ICP-MS. The oven-dried samples, consisting of three replicates of 15 plants each from the control treatment (water) and GR-MOF-27 at 91 ppm, were weighed, ground into powder, digested with 4 mL of HNO_3 during sonication and the resulting solution was then adjusted to a final volume of 50 mL. The values obtained were normalized to the dried weight per plant to allow comparison between samples. The different groups have the following Ca, Mg, and P content. Control: Ca 2.03 ± 0.12, Mg 1.28 ± 0.06, and P 1.92 ± 0.07; GR-MOF-27 91 ppm: Ca 1.86 ± 0.12, Mg 3.66 ± 0.86, P 4.49 ± 1.01; GR-MOF-27: Ca 1.76 ± 0.21, Mg 1.99 ± 0.59, and P 2.59 ± 0.73. A significant increase in Mg and P content was observed in plants treated with 91 ppm of GR-MOF-27 (*p*-value <0.01), whereas treatment with 910 ppm did not result in a significant increase compared to the control samples. Therefore, plants treated with 91 ppm of GR-MOF-27 showed an increase in Mg and P content by 64.9 and 57.4%, respectively, compared to the control.

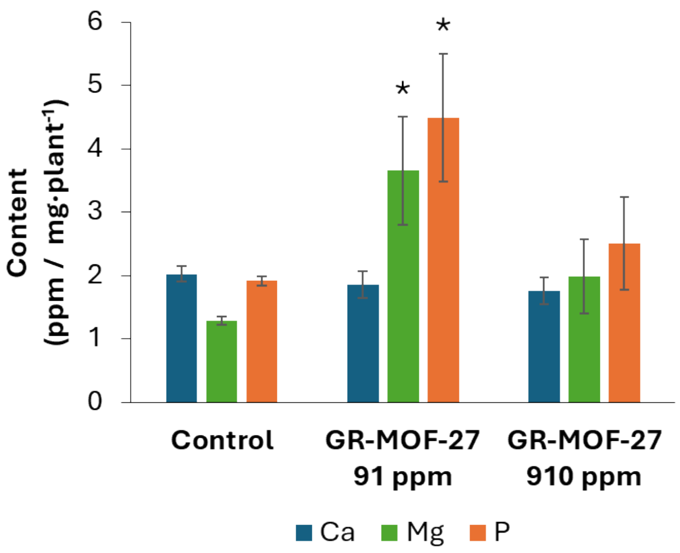

Figure S11. Ca, Mg, and P content (ppm) relative to the dried weight (mg) per plant treated with water (control), 91 and 910 ppm of GR-MOF-27. Average and standard deviation is represented. Values of each treatment were compared to identify significant differences; ANOVA test was performed where *p*-value *< 0.01.

S5. References

(1) Bruker AXS Inc.: Madison. Burker Apex2. B. A. I. Bruker Apex2. WI, USA 2004.

(2) Sheldrick, G. M. SADABS 1996, Program for Empirical Adsorption Correction. 1996, p Available online: https://cmacd.myweb.cs. uwindsor.

(3) Sheldrick, G. M. SHELXT - Integrated Space-Group and Crystal-Structure Determination. *Acta Crystallogr A* **2015**, *71* (1), 3–8. https://doi.org/10.1107/S2053273314026370.

(4) Dolomanov, O. V.; Bourhis, L. J.; Gildea, R. J.; Howard, J. A. K.; Puschmann, H. OLEX2: A Complete Structure Solution, Refinement and Analysis Program. *J Appl Crystallogr* **2009**, *42* (2), 339–341. https://doi.org/10.1107/S0021889808042726.

(5) Gautam, S.; Lakhanpal, I.; Sonowal, L.; Goyal, N. Recent Advances in Targeted Drug Delivery Using Metal-Organic Frameworks: Toxicity and Release Kinetics. *Next Nanotechnology* **2023**, *3–4*, 100027. https://doi.org/10.1016/j.nxnano.2023.100027.

(6) Plazinski, W.; Rudzinski, W.; Plazinska, A. Theoretical Models of Sorption Kinetics Including a Surface Reaction Mechanism: A Review. *Advances in Colloid and Interface Science*. November 30, 2009, pp 2–13. https://doi.org/10.1016/j.cis.2009.07.009.

(7) Higuchi, W. I. Analysis of Data on Medicament Release Form Ointments. *J Pharm Sci* **1962**, *51*, 802–804.

(8) Dash, S.; Murthy, P. N.; Nath, L.; Chowdhury, P. Kinetic Modeling on Drug Release from Controlled Drug Delivery Systems. *Acta Pol Pharm* **2010**, *67*, 217–223. https://doi.org/10.1016/S0928-0987(01)00095-1.

(9) Zhang, W.; Liu, Y.; Muneer, M. A.; Jin, D.; Zhang, H.; Cai, Y.; Ma, C.; Wang, C.; Chen, X.; Huang, C.; Tang, Y.; Wu, L. Characterization of Different Magnesium Fertilizers and Their Effect on Yield and Quality of Soybean and Pomelo. *Agronomy* **2022**, *12* (12), 2–18. https://doi.org/10.3390/agronomy12112693.

(10) Elsabagh, S. S.; Elkhatib, E. A.; Rashad, M. Eco-Friendly Nano-Enabled Fertilizers Derived from Date Industry Waste for Sustainable and Controlled-Release of P, K and Mg Nutrients: Sorption Mechanisms, Controlled-Release Performance and Kinetics. *Bioresour Bioprocess* **2024**, *11* (1). https://doi.org/10.1186/s40643-023-00716-6.

(11) Geng, G.; Ye, X.; Ren, T.; Zhang, Y.; Li, X.; Cong, R.; Cakmak, I.; Lu, Z.; Lu, J. Optimal Magnesium Management for Better Seed Yield and Quality of Rapeseed Based on Native Soil Magnesium Supply. *European Journal of Agronomy* **2024**, *161*. https://doi.org/10.1016/j.eja.2024.127364.

(12) Qu, S.; Li, H.; Zhang, X.; Gao, J.; Ma, R.; Ma, L.; Ma, J. Effects of Magnesium Imbalance on Root Growth and Nutrient Absorption in Different Genotypes of Vegetable Crops. *Plants* **2023**, *12* (20). https://doi.org/10.3390/plants12203518.
